# Development of a Mouse Monoclonal Antibody Cocktail for Post-exposure Rabies Prophylaxis in Humans

**DOI:** 10.1371/journal.pntd.0000542

**Published:** 2009-11-03

**Authors:** Thomas Müller, Bernhard Dietzschold, Hildegund Ertl, Anthony R. Fooks, Conrad Freuling, Christine Fehlner-Gardiner, Jeannette Kliemt, Francois X. Meslin, Charles E. Rupprecht, Noël Tordo, Alexander I. Wanderler, Marie Paule Kieny

**Affiliations:** 1 WHO Collaborating Centre for Rabies Surveillance and Research, Friedrich-Loeffler-Institute, Federal Research Institute for Animal Health, Wusterhausen, Germany; 2 WHO Collaborating Centre for Neurovirology, Department of Microbiology and Immunology, Thomas Jefferson University, Philadelphia, Pennsylvania, United States of America; 3 WHO Collaborating Centre for Reference and Research on Rabies, Wistar Institute, Philadelphia, Pennsylvania, United States of America; 4 WHO Collaborating Centre for the Characterization of Rabies and Rabies-related Viruses, Veterinary Laboratories Agency, Department of Virology, New Haw, Addlestone, Surrey, United Kingdom; 5 WHO Collaborating Centre for Rabies Control, Pathogenesis and Epidemiology in Carnivores, Canadian Food Inspection Agency (CFIA) Centre of Expertise for Rabies, Ottawa, Ontario, Canada; 6 Neglected Zoonotic Diseases (NZD), Department of Neglected Tropical Diseases (NTD), Cluster HIV/AIDS, Malaria, Tuberculosis and Neglected Tropical Diseases (HTM), World Health Organization, Geneva, Switzerland; 7 WHO Collaborating Centre for Reference and Research on Rabies, Rabies Section, Division of Viral and Rickettsial Diseases, Viral and Rickettsial Zoonoses Branch, National Center for Infectious Diseases, Centers for Disease Control and Prevention, Atlanta, Georgia, United States of America; 8 Unit Antiviral Strategy, CNRS URA-3015, Institut Pasteur, Rabies Unit, Paris, France; 9 Initiative for Vaccine Research, Vaccines & Biologicals, Health Technology & Pharmaceuticals, World Health Organization, Geneva, Switzerland; Swiss Tropical Institute, Switzerland

## Abstract

As the demand for rabies post-exposure prophylaxis (PEP) treatments has increased exponentially in recent years, the limited supply of human and equine rabies immunoglobulin (HRIG and ERIG) has failed to provide the required passive immune component in PEP in countries where canine rabies is endemic. Replacement of HRIG and ERIG with a potentially cheaper and efficacious alternative biological for treatment of rabies in humans, therefore, remains a high priority. In this study, we set out to assess a mouse monoclonal antibody (MoMAb) cocktail with the ultimate goal to develop a product at the lowest possible cost that can be used in developing countries as a replacement for RIG in PEP. Five MoMAbs, E559.9.14, 1112-1, 62-71-3, M727-5-1, and M777-16-3, were selected from available panels based on stringent criteria, such as biological activity, neutralizing potency, binding specificity, spectrum of neutralization of lyssaviruses, and history of each hybridoma. Four of these MoMAbs recognize epitopes in antigenic site II and one recognizes an epitope in antigenic site III on the rabies virus (RABV) glycoprotein, as determined by nucleotide sequence analysis of the glycoprotein gene of unique MoMAb neutralization-escape mutants. The MoMAbs were produced under Good Laboratory Practice (GLP) conditions. Unique combinations (cocktails) were prepared, using different concentrations of the MoMAbs that were capable of targeting non-overlapping epitopes of antigenic sites II and III. Blind *in vitro* efficacy studies showed the MoMab cocktails neutralized a broad spectrum of lyssaviruses except for lyssaviruses belonging to phylogroups II and III. *In vivo*, MoMAb cocktails resulted in protection as a component of PEP that was comparable to HRIG. In conclusion, all three novel combinations of MoMAbs were shown to have equal efficacy to HRIG and therefore could be considered a potentially less expensive alternative biological agent for use in PEP and prevention of rabies in humans.

## Author Summary

Human mortality from endemic canine rabies is estimated to be 55,000 deaths per year in Africa and Asia, yet rabies remains a neglected disease throughout most of these countries. More than 99% of human rabies cases are caused by infections resulting from a dog-bite injury. In the vast majority of human exposures to rabies, patients require post-exposure prophylaxis (PEP), which includes both passive (rabies immunoglobulin, RIG) and active immunization (rabies vaccine). The number of victims requiring PEP has increased exponentially in recent years, and human and equine RIG (HRIG and ERIG) were not sufficiently available in countries where canine rabies is endemic. Rabies virus-neutralizing monoclonal antibodies (MAbs) of mouse (Mo) origin have been identified as promising alternatives to HRIG and ERIG. We have developed and assessed both *in vitro* and *in vivo* unique mouse monoclonal antibody (MoMAb) cocktails, which are highly efficacious. Three novel combinations were shown to have an equal or superior efficacy to HRIG and therefore could be considered a potentially less expensive alternative for passive prophylactic use to prevent the development of rabies in humans, particularly where needed most in developing countries.

## Introduction

Rabies is an acute viral encephalomyelitis in humans and other warm-blooded vertebrates, caused by a member of the genus *Lyssavirus* of the *Rhabdoviridae* family. Within the genus, seven genotypes (gts) have been delineated and the classification for another four recently found viruses within the genus is still pending.

Lyssavirus Gts have been further segregated into phylogroups on the basis of their glycoprotein gene sequence, and the pathogenicity and immunogenicity of the virus. The prototype virus of the genus is rabies virus (RABV; gt 1), which along with Duvenhage virus (DUVV; gt 4), European bat lyssavirus type-1 and -2 (EBLV-1 and -2; gts 5 and 6, respectively), belongs to phylogroup I [1]. The unclassified lyssaviruses Aravan virus (ARAV), Khujand virus (KHUV) and Irkut virus (IRKV) also cluster with this group [2]. The African gts, Lagos bat virus (LBV; gt 2) and Mokola virus (MOKV; gt 3) were assigned to phylogroup II [1]. Studies have shown that West Caucasian Bat virus (WCBV) is the most divergent member of the genus and may not belong to either phylogroup I or II but rather represents a new phylogroup III [2],[3].

Classical rabies caused by the prototype RABV is the most important public health problem world-wide. Only certain countries e.g. the United Kingdom, New Zealand, the state of Hawaii (USA), Australia and Antarctica and parts of Western Europe, are currently free of the virus, either historically or through successful rabies elimination programs. The epidemiology of this enzootic disease in rabies endemic countries is characterized by the principal reservoir host species in which the virus circulates. Two broad circulation patterns are recognized: sylvatic rabies (involving wildlife in both *carnivora* and *chiroptera* orders) and canine rabies, which represents the heaviest burden on human health. The occurrence of these two circulation patterns follows a general geographic and socio-economic pattern [4]. Canine rabies causes an estimated 55,000 human deaths each year, especially in Asia and Africa, although the true burden of the disease is unknown due to underreporting and poor surveillance systems in many areas of the world [5]–[7]. It has been estimated that half of the world's population live in a canine rabies-endemic area [8]. Although the most efficient way of preventing human rabies cases is the control of the disease in the vector population by mass dog vaccination combined with population control, such efforts have not been taken systematically in large parts of Africa and Asia. Also effective vaccines that protect humans against rabies are not universally available throughout the world. The largest number of fatalities is reported in under-privileged children principally those under 14 years of age that live in the poorer countries of the world. In greater than 99% of cases, human death results from dog-bite injury [8]. In the majority of cases, a category 3 exposure occurs, which includes bites and/or contamination of mucous membranes with saliva containing the virus. In a rabies infected area, a category 3 exposure should be treated immediately by wound treatment (thorough washing) plus the administration of rabies post-exposure prophylaxis (PEP) comprised of both rabies immunoglobulin (RIG) for passive protection and rabies vaccine to induce circulating virus-neutralizing antibodies (VNAs) [4]. Evidence suggests that when PEP is administered in a timely manner RABV is cleared before it enters the CNS [9]. The mode of protection is likely to be virus neutralization by antibodies or antibody-mediated clearance of virus-infected cells [10]–[12].

Currently, human and equine polyclonal anti-rabies immune globulin (HRIG and ERIG, respectively) are used in passive immunization. They are prepared from pooled sera taken from hyper immunized humans or horses, respectively [13]. HRIG is available in limited quantities on specific markets and is prohibitively expensive (approximately US$250 per adult treatment) for most rabies virus-exposed humans living in developing countries. The cost is approximately five times that of purified horse serum. ERIG although being a potent biological may show significant differences in adverse reaction rates, reflecting differing manufacturing or purification processes and protein content and therefore may lead to complications such as serum sickness or anaphylactic shock [13]. However, when modern purified ERIG is used the prevalence of anaphylaxis and mild serum sickness-like reactions is very low [14],[15].

Other problems arising from the production of both HRIG and ERIG include high manufacturing costs and the potential risk contamination in human blood products including unknown agents and pathogens [16]. In addition, animal protection groups that are becoming more influential in developing countries are trying to stop animal usage for production of antisera.

Thus, besides efforts to improve the supply with HRIG and ERIG, accelerated research and development of alternative products are required and essential for the future of global health practices in the management of human rabies. Monoclonal antibodies (MAbs) are particularly attractive alternatives for HRIG and ERIG and could be a keystone in rabies prevention as they seem to represent a revolution in clinical medicine [17]. Mabs have also been approved to treat cancer, inflammatory and other infectious diseases and to prevent graft rejection [18],[19]. Anti-RABV glycoprotein (G) MAbs are considered alternatives to HRIG and ERIG because they would be safer products for use in PEP preparations for humans [4].

Mouse and human MAbs (MoMAbs and HuMAbs) that neutralize RABV have been produced by different groups of investigators [10]–[12], [20]–[26]. Both MAb types could form the basis for viable alternative strategies for PEP in humans as they have many advantages over HRIG and ERIG. Ideally, a collection of MAbs capable of neutralizing all RABV strains relevant to human rabies would be required [16]. Till now, despite the identification of numerous potential HuMAbs for rabies PEP, only one HuMAb cocktail comprising two HuMAbs has been developed, thoroughly characterized both *in vitro* and *in vivo* and successfully clinically tested in two phase I studies [27]–[29].

Although MoMAbs have been used extensively for antigenic typing of RABV strains and their protective activity has been demonstrated in certain animal models [10]–[12],[25], a unique MoMAb cocktail combination to replace HRIG or ERIG has not yet been developed. The objective of this WHO co-ordinated project has been to evaluate existing MoMAbs at WHO Collaborating Centres for their capacity to neutralize a variety of RABVs, principally canid strains. The most promising of these were then tested in combinations of a minimum of two anti-G MuMAbs, targeting distinct antigenic sites, to replace HRIG and ERIG for human PEP against rabies. Here we report three unique MoMAb cocktail combinations that would be suitable replacements for HRIG and ERIG, based on the stringent criteria for each of the selected MoMAbs concerning neutralizing potency, binding specificity, and spectrum of neutralization of ‘street’ RABV isolates. The final characterisation of these three unique MoMAb cocktail combinations was based on their cross-reactivity against RABVs *in vitro* and efficacy *in vivo*, in protecting animals against rabies.

## Materials and Methods

### Selection of MoMAbs and technical information

Panels of well-defined neutralizing anti-G MoMAbs available from four WHO collaborating centres for rabies were screened for their suitability as potential candidate MoMAbs using the following selection criteria:

1. Biological activity–They should exhibit (i) a minimum neutralizing potency of 100 IU/ml of crude hybridoma supernatant, (ii) a consistent production stability (loss of MoMAb secretion should not exceed 10% up to 30 passages), (iii) a broad spectrum of reactivity with regard to genotype I (canid strains);
2. Binding specificity–They should target distinct, non-overlapping epitopes (antigenic sites I–III) on the RABV G;
3. Immunoglobulin isotype (Ig)–They should be preferably of isotype IgG1, 2a or 3;
4. History of hybridomas–There should be sufficient background information on the relative risk of possible contamination with transmissible spongiform encephalitis (TSE) agents and on the regional sources of known batches of fetal calf serum used for hybridoma growth.

Further technical information on the recommended culture conditions for hybridomas, available nucleotide sequence of heavy and light chain cDNAs, and intellectual property rights issues were also established (Table 1). In one case, a candidate MoMAb had to be excluded from further consideration because of intellectual property ownership; however, the MoMAb was still included in the study for comparative purposes.

**Table 1:** Available technical information for candidate MoMAbs.

History of hybridomas	E559.9.14	1112-1	62-7-13	M727-5-1	M777-16-3
Mouse strain providing B-cells	BALB/c mice	BALB/c mice	BALB/c mice	BALB/c mice	BALB/c mice
Antigen	ERA G protein	ERA G protein	whole ERA	whole ERA, #167–169	whole ERA, #167–169
Fusion partner (Year of fusion)	P3-X63Ag8 (1979)	653 (1985)	Sp2/0–Ag14 myeloma (1983)	Sp2/0–Ag14 myeloma (1994)	Sp2/0–Ag14 myeloma (1994)
Reference	[35]	[50]	no	no	no
Number of cloning steps	4	Not known	3	4	4
Purity/homogeneity of cell line	Not known	Not known	Sub-cloned 2x, single IgG peak	isotype as pure IgG 2a	isotype as pure IgG 1
Origin of FCS used	New Zealand	USA	USA (GIBCO)	USA (Sigma), Canada (Wisent)	USA (Sigma), Canada (Wisent)
Absence of adventitious agents	Mycoplasma free	n.d.	Per WHO screening request	n.d.	n.d.
**Culture conditions**					
Medium	Iscove's DMEM 1	DMEM (modified)	Iscove's DMEM 2	HY-HT (10% FCS)	HY-HT (10% FCS)
Cell concentration	10^4^–10^6^	10^4^–10^6^	2×10^5^	6×10^4^−3×10^5^	7×10^4^−3×10^5^
Serum-free culture medium	CD HM or PFHM II protein-free	Not tested	tested but no specification	Ultradoma-PF	Ultradoma-PF
**Type of immunoglobulin**					
IgG subtytpe	IgG 1 (ELISA)	IgG 1 (ELISA)	IgG 2b (ELISA)	IgG2a (FCA)	IgG 1 (FCA)
Heavy/light chains cDNAs	Yes	Yes	no	no	no
Antigenic site recognized on G	II	II c	III	II	II
Method for determining epitope	sequencing	sequencing	cross-neutralisation	cross-neutralisation	cross-neutralisation
**Escape mutants**					
derivation	SAD B19	CVS-11	not available	not available	ERA
aa substitutions in G	aa 57 (Leu to Arg)	aa 53 (Gly to Glu)			aa 198 (Lys to Glu)
	aa 217 (Lys to Glu)				aa 286 (Ala to Thr)
**Production yield**					
Yield in IU/ml (crude hybridoma)	62.5	3	30–60	22–32	11–32

Legend: aa–amino acid, CVS 11–Challenge virus standard 11, DMEM–Dulbeccos' minimum essential medium, ELISA–enzyme linked immunosorbent assay, ERA–Evelyn Rokitniki Abelseth SAD derived RABV strain, FCA–Flouricon-CA Assay, HB–hybridization medium, SAD–Street Alabama Dufferin strain of RABV. Media specification: Iscove's DMEM 1 = Iscove's modified DMEM + HAM F12 (1∶1) + 10% FCS; Iscove's DMEM 2 = Iscove's modified DMEM + ITS + antibiotics/antimycotics + L-glutamine + 5% FCS.

The minimum neutralizing potency of the crude hybridoma supernatant and the relative stability of antibody produced, in terms of its virus-neutralizing activity was determined under both serum-containing and serum-free conditions using the rapid fluorescent focus inhibition (RFFIT) test and the fluorescent antibody virus neutralization (FAVN) test as described [30],[31]. The IgG isotype or subtype of the MuMAbs was determined by a commercially available dipstick typing test kit (Serotec, Düsseldorf, Germany) or an in-house-developed Fluoricon assay. Briefly, polystyrene beads (IDEXX, Westbrook, USA) were coated with unlabelled Ig (IgM+IgG+IgA, H and L chains; Southern Biotech, Birmingham, USA), incubated in Fluoricon assay plates (IDEXX) with hybridoma supernatant, washed and then incubated with the FITC-labelled isotype-specific goat-anti-mouse antibodies (Southern Biotech). Unbound, labelled antibody was washed off and then the plates were read in a Fluorescence Concentration Analyzer (FCA, IDEXX).

The binding specificity of MoMAb candidates, determined by the localization of the potential binding site on the virus G, was assessed using two different approaches, either by generating MoMAb-specific escape mutants or *in vitro* cross-neutralisation assays. To generate MoMAb-specific escape mutants, fixed RABV strains (SAD, CVS, ERA) were propagated in BHK-21 cells in serial passages, at a multiplicity of infection of 0.01, in the presence of a MoMAb at a sufficiently low concentration to have no neutralizing effect. Subsequent sequence analysis of the G gene of derived virus escape mutants and multiple alignments were undertaken as described [32]. MoMAbs that did not generate any escape mutants were checked for their neutralizing potency with escape mutants derived from other MoMAb candidates or from MAb D1 (Institut Pasteur, Paris, France), which is a well-characterised antibody with a previously identified binding specificity to binding site III on the RABV G [26],[33],[34].

### *In vitro* neutralization studies

In a preliminary study, MoMAbs, either purified or in the form of crude cell culture supernatant, were tested *in vitro* for their broad spectrum of reactivity. In a second experiment, based on the selection criteria in terms of binding specificity and broad spectrum cross-reactivity, blinded mini-cocktails comprising of two MoMAbs targeting different epitopes, e.g. binding sites, on the RABV G, were assessed further under the same conditions (see below). Testing of the *in-vitro* broad spectrum cross-reactivity of the selected MoMAb candidates as well as representatives of all the known lyssavirus gts (gt 1–7, n = 28) was undertaken in three independent laboratories: Friedrich-Loeffler-Insitute (FLI, Germany), Centers for Disease Control and Prevention (CDC, USA), and Canadian Food Inspection Agency (CFIA, Canada), as described [23],[35]. Putative lyssavirus genotypes (Aravan virus–ARAV, Khujand virus–KHUV, Irkut virus–IRKV, and West Caucasian Bat virus–WCBV) were also assessed (n = 4). The principal focus was on canid strains of RABV (gt 1) (N = 20) from specific host species and geographical areas across the world [4]. Prior to testing, monolayers of murine neuroblastoma cells (NA 42/13) were infected with selected lyssavirus field strains, at a multiplicity of infection of 0.1, for 1 h at 37°C in 5% CO_2_. Subsequently, the virus inoculum was removed and fresh Minimal Essential Medium (MEM) was added to the cells. Following incubation for 72 h at 37°C in 5% CO_2_, cell culture supernatants were collected and titrated on BHK-21 or MNA cells (BioWhitaker, Walkersville, USA). Up to three passages were undertaken to obtain sufficient virus titres. Viruses were stored at −80°C until further use. The neutralizing potency of the MoMAbs was determined by RFFIT or FAVN using BHK-21 or MNA cells infected with a constant amount of virus and varying amounts of the MAb (endpoint titration) as described [23],[36]. Briefly, MoMAbs and/or blinded mini-cocktails were serially diluted and incubated with 10^2^ or 10^4^ FFU/ml of selected lyssavirus strains for 24 h. Subsequently, virus growth was detected by fixing the cells with cold 75% acetone and then staining with a fluorescein isothiocyanate (FITC)-labelled anti-rabies conjugate. VNA titres were expressed as the reciprocal of the dilution at which 50% of the wells showed complete neutralization of virus growth. The titres were compared to those of an international standard rabies immunoglobulin (SRIG, 2nd human rabies immunoglobulin preparation, National Institute for Biological Standards and Control, Potters Bar, UK) adjusted to 30 IU/ml and converted into international units per ml (IU/ml).

### Batch production and testing of candidate MoMAbs under GLP conditions

A Master Cell Bank was prepared for each candidate hybridoma by the National Institute for Biological Standards and Control (NIBSC, UK). A vial corresponding to each of the selected hybridomas was provided to a service manufacturer (Apotech, Lausanne, Switzerland) contracted by WHO for production of MuMAbs under Good Laboratory Practice (GLP) conditions in small-scale cultures using culture media as recommended (Table 1). Subsequently, MoMAbs were purified by Protein A affinity chromatography essentially as described [16]. The purity of all MoMAb preparations was assessed by electrophoresis through a 12.5% polyacrylamide gel under reducing conditions (SDS-PAGE) and subsequent Coomassie blue staining. Yields were expressed in protein mass (mg/L). VNA titres (IU/L) of the purified MoMAbs were determined by RFFIT in three independent assays using CSV-11 as a challenge virus as described [37]. Unique standardized cocktail combinations consisting of two purified MoMAbs of equal concentrations (1∶1) and targeting non-overlapping epitopes (in different antigenic sites) were prepared for blind *in vivo* testing in parallel with HRIG as a positive control. For this purpose, the volume (ml) delivering 1000 IU for each MoMAb was determined, and mixed with buffer to the desired final concentration. Two sets of 1∶1 MoMAb cocktails were theoretically adjusted to a total of either 2000 IU per 5 ml or 2000 IU per 10 ml equalling 400 and 200 IU per ml, respectively. The latter was also simultaneously blind tested *in vitro* as described above but with an incubation period of at least 48 h to improve robustness of the data.

### *In vivo* testing

*In vivo* testing was undertaken as a ”down selection” as described [11],[38]. Briefly, 10 female Syrian hamsters in each group were inoculated with 0.05 ml of RABV virus (Mexican, Thai, or Indian canine RABV variant) intramuscularly (i.m.) in the gastrocnemius muscle. Six or 24 hours later, animals were given biologics or PBS (negative control). Undiluted commercial HRIG (Sanofi-Aventis, 150 IU/ml) or candidate MoMAbs were administered i.m. in the gastrocnemius muscles in volumes of 50 µl. All i.m. injections were undertaken using a tuberculin syringe and needle not exceeding 23 gauge. After challenge, animals were observed twice daily and euthanized at the first clinical signs of rabies (eg. paresis, paralysis, aggression). Brain tissue was harvested to confirm the rabies infection using the direct fluorescent antibody (DFA) test [39]. All animals surviving up to 30 days post infection were euthanized and tested for rabies as described above. Animal-handling and experimental procedures were undertaken in compliance with the CDC's Institutional Animal Care and Use Committee (IACUC) guidelines. Ethical approval was obtained for each study before experiments were initiated (IACUC CDC, USA).

## Results

### Selection and further characterization of MoMAbs

Five candidate anti-G MoMAbs, E559.9.14 from FLI, Germany, 1112-1 from the Wistar Institute, USA, 62-71-3 from the CDC, USA, M727-5-1 and M777-16-3 from CFIA, Canada met the selection criteria and were short-listed to be included in the cocktail and subjected to further investigation. All hybridomas were derived from B cells of BALB/c mice immunized intraperitoneally (i.p.) with either purified G or whole virus antigen of the ERA vaccine strain of RABV. The hybridomas were generated with three different fusion partners and at least three cloning steps (Table 1). Based on information of the cell culture history for each MoMAb, only approved fetal calf serum originating from countries being free of foot and mouth disease (FMD) and TSE, was used in the MAb production.

Candidate MoMAbs were shown to represent two different subtypes of immunoglobulin, i.e. IgG 1 and IgG 2, as determined by ELISA or FCA. Sequence analysis of the G gene of the SAD B19-, CVS-11- and ERA-derived escape mutants of MoMAbs E559.9.14, 1112-1, and M777-16-3 showed one or as many as two amino acid substitutions in the virus G compared to the original wildtype viruses (Table 1). *In vitro* cross-neutralisation assays performed with the escape mutant of MoMAb E559.9.14 showed that all but one MoMAb recognized epitopes at antigenic site II on the RABV G. MoMAb 62-7-13 was the only antibody showing a reaction pattern similar to MoMAb D1, which is known to recognize conformational epitopes of antigenic site III on G trimers (Table 2).

**Table 2:** Neutralization pattern of candidate MoMAbs (E559.9.14, 62-7-13, 727-5, 777-16) or other MoMAbs (1112-1, D1) with RABV (E559.9.14 antigenic site II escape mutant, 10^4^ FFU/ml) under varying VNA titres (IU/ml).

	Titre, IU/ml								
MAb	10	5	2	1	0.5	0.25	0.125	0.063	0.03
E559.9.14	+	+	+	+	+	+	+	+	+
62-7-13	−	−	−	−	−	−	−	−	+
727-5	+	+	+	+	+	+	+	+	+
777-16	+	+	+	+	+	+	+	+	+
1112-1	+	+	+	+	+	+	+	+	+
D1	−	−	−	−	−	−	−	−	−

Absence (−) and presence (+) of viable virus is indicated.

The production stability of candidate MoMAbs was determined after 30 cell passages in serum-containing medium. Only slight instabilities were observed under laboratory conditions with a few candidate MoMAbs, but the loss of antibody secretion was less than 10%, as required if at all (Table 1). Adaptation to serum-free conditions resulted in a considerable decrease of MoMAb production. For MuMAb E559.9.14., for example, VNA titres dropped to 16, 32, and 64 IU/ml when harvested at day 3, 6 and 10, respectively, however, the production remained stable at a lower level for up to 30 cell passages. Under serum-free medium conditions, MoMAb M727-5-1 and MoMAb M777-16-3 VNA titres fluctuated from 22.63, 22.63, 45.25, 32.0 and 32.0 IU/ml to 32.0, 16.0, 45.25, 11.31 and 22.63 IU/ml after 5, 11, 15, 20 and 25 cell passages, respectively. Further characterization of individual candidate MoMAbs is summarized in Table 1. MoMAb 1112-1 had to be excluded from further consideration because of proprietary issues but was kept in the study for comparison.

### *In vitro* studies

A broad *in vitro* cross-reactivity of the candidate MoMAbs with RABV and several of the other lyssaviruses was demonstrated at different virus doses. None of the MoMAbs completely neutralized the full spectrum of lyssaviruses tested. All five candidate MoMAbs as well as standard RIG (SRIG) did not neutralize MOKV (gt 3) and WCBV (putative gt). Also, MoMAb 62-71-3 failed to recognize DUVV (gt 4), EBLV-1 (gt 5), and MoMAb 1112-1 did not recognize1 lyssavirus (KUHV–putative gt) (Table 3). None of them recognized LBV. As regards RABVs (gt1), the number of gt1 RABVs the candidate MoMAbs failed to neutralize ranged between 2 and 7 (of the 20 RABVs tested). All but one (MoMAb 62-7-13) failed to recognize the Kelev strain of RABV and a skunk RABV variant originating from California, USA (Table 3). MoMAbs M727-5-1 and M777-16 required minimally higher concentrations for neutralization compared to the other candidate MoMAbs. Interestingly, a cocktail comprising all five candidate MoMAbs was able to neutralize all viruses tested except MOKV and WCBV. Identical results were obtained with the polyclonal SRIG using the same concentrations (data not shown).

**Table 3:** *In vitro* neutralization pattern of individual candidate MAbs.

Lyssaviruses	gt	Virus dose (log10)	SRIG	M777-16	M727-5	62-7-13 (03-043)	62-7-13 (03-041)	62-7-13 (03-026)	1112-1	E559
Bobcat, USA	1	4	0.125	1.0	5.0	0.063	n.d.	n.d.	0.25	+
CVS-11	1	4	0.063	2.0	5.0	0.125	n.d.	n.d.	0.25	0.25
Dog, Azerbaizhan	1	4	0.25	+	+	0.25	n.d.	n.d.	1.0	10.0
Dog, Ethiopia	1	4	0.125	5.0	5.0	0.5	n.d.	n.d.	0.5	0.5
Dog, India	1	4	1.0	5.0	+	0.125	n.d.	n.d.	1.0	0.25
Dog, Mexico	1	4	0.5	10.0	+	0.5	n.d.	n.d.	2.0	0.063
Dog, Nepal	1	4	0.25	2.0	10.0	0.125	n.d.	n.d.	0.125	0.125
Dog, Turkey	1	4	0.125	+	+	0.125	n.d.	n.d.	+	0.5
Fox, Eastern Europe	1	4	0.5	1.0	5.0	0.125	n.d.	n.d.	0.25	0.125
Fox, Europe	1	4	0.125	1.0	5.0	5.0	n.d.	n.d.	0.5	0.25
Kelev, Israel	1	4	0.25	+	+	0.063	n.d.	n.d.	+	+
Polar fox, Norway	1	4	0.5	10.0	10.0	0.5	n.d.	n.d.	0.5	0.25
PV	1	4	0.25	2.0	10.0	+	n.d.	n.d.	0.5	0.063
SAD B19	1	4	0.25	5.0	10.0	2.0	n.d.	n.d.	0.5	0.25
Wolf, Bosnia	1	4	0.125	2.0	+	0.125	n.d.	n.d.	0.125	0.25
EBLV-1, Germany	5	4	1.0	1.0	10.0	+	n.d.	n.d.	0.25	0.5
EBLV-2, UK	6	4	2.0	5.0	10.0	0.125	n.d.	n.d.	0.5	0.25
Arctic fox, AK, USA	1	2	0.016	0.014	0.016	0.019	0.114	0.012	0.0136	0.016
CVS-11	1	2	0.016	0.014	0.016	0.019	0.114	0.012	0.0136	0.016
CVS-11	1	2	0.016	0.014	0.016	0.019	0.002	0.002	0.0136	0.016
Gray fox, TX, USA	1	2	0.016	0.014	0.016	0.003	0.114	0.002	0.068	0.016
Raccoon, SC, USA	1	2	0.08	0.014	0.08	0.003	0.0572	0.012	+	0.08
Skunk, CA, USA	1	2	0.08	+	+	0.003	0.002	0.002	+	+
Skunk, SC, USA	1	2	0.016	0.014	0.016	+	+	+	+	0.016
MOKV, Africa	3	2	+	+	+	+	+	+	+	+
DUVV, Africa	4	2	0.08	0.014	0.016	+	+	+	0.068	0.016
EBLV-1, Europe	5	2	0.08	0.014	0.016	+	+	+	0.0136	0.016
EBLV-2, Europe	6	2	0.08	0.014	0.016	0.003	0.002	0.002	0.0136	0.016
ABLV, Australia	7	2	0.016	0.014	0.016	0.003	0.114	0.012	0.068	0.016
ARAV, Asia		2	0.08	0.014	0.08	0.019	0.002	0.002	0.34	0.016
IRKV, Asia		2	0.08	0.014	0.016	0.019	0.114	0.002	0.068	0.016
KHUV, Asia		2	0.08	0.014	0.016	0.003	0.002	0.002	+	0.016
WCBV, Europe		2	+	+	+	+	+	+	+	+

Tests were conducted in comparison to standard rabies immunoglobulin (SRIG) against lyssa- (gt 1–7) and putative lyssavirus gts. MoMAbs M777-16 and M727-5 were used purified and the remaining as cell culture supernatants. For MAb 62-7-13, three different harvests were tested. Figures in boxes show the minimum MoMAb concentration in IU/ml at which complete neutralization was observed. Boxes with cross (+) represent presence of viable virus.

### Batch production and testing of candidate MoMAbs under GLP conditions

**Figure 1:**
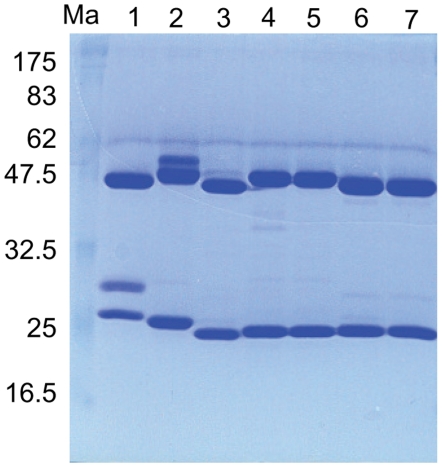
Coomassie blue staining of purified MoMabs (5 µg/well) demonstrating appropriate size of light chain and heavy chain. Ma–molecular weight marker in kD; lane1, E559 (batch # 603-02); lane 2, 62-7-13 (batch # 604-26); lane 3, 1112-1 (batch # 604-26); lane 4, M777-16-3 (batch # 605-03); lane 5, M777-16-3 (batch # 605-12); lane 6, M727-5-1 (batch # 605-03); lane 7, M727-5-1 (batch # 605-19).

The minimum yields for purified MoMAbs obtained under GLP conditions in small-scale cultures were 15 (1112-1, M725-1), 20 (E559.9.14), 25 (62-7-13) and 40 (M777-16-3) mg/L. All purified MoMAbs produced under these conditions showed two major bands, at 47 and 20 to 25 kDa on SDS-PAGE corresponding to isolated heavy and light immunoglobulin chains (Figure 1). The geometric mean VNA titres of purified candidate MoMAbs (1 mg/ml) varied from 474 to 10,257 IU/ml. Based on these data, the immunoglobulin titres (total yield of supernatants) for the five MoMAb hybridomas was estimated to range between 9,480 and 153,855 IU/L (Table 4).

**Table 4:** Neutralization results obtained after batch production under GLP conditions.

	Purified MoMAbs						Supernatant
MoMAbs	Antigen content (g/ml)	GEO VNA (IU/ml)	SD	Min	Max	VNA (IU/mg)	VNA (IU/mg)
E559.9.4	1	474.29	86.39	375.04	532.58	474	>9,480
1112-1	1	5990.38	222.13	5860.26	6246.87	5990	>89,850
62-7-13	1	501.28	251.30	337.38	790.61	501	>12,525
M727-5-1	1	10256.79	2088.87	8482.00	12558.75	10257	>153,855
M777-16-3	1	1962.40	511.87	1534.12	2529.31	1962	>78,480
**Controls**							
WHO SRIG		0.50	0.00	0.50	0.50		
Negative control		0.08	0.00	0.07	0.08		

Geometric mean (GEO) VNA titres and standard deviation (SD) of 1 mg/ml of purified MoMAbs as determined by RFFIT in three independent tests and subsequent estimation of the yield of supernatant of the five hybridomas in comparison to a negative control and the WHO standard rabies immunoglobulin (SRIG).

### *In vivo* and *in vitro* studies of 1∶1 MoMAb mini-cocktail formulations

The capacity of the 4 MoMAbs to recognize antigenic site II and the 1 MoMAb to recognize antigenic site III (Table 1) dictated the preparation of unique 1∶1 MoMAb cocktail combinations targeting non-overlapping epitopes. In particular, MoMAb 62-7-13 (antigenic site III) was combined with each of the four remaining MoMAbs (antigenic site II). MoMAb cocktail combinations 62-7-13/62-7-13, 62-7-13/1112-1 and MoMAb 62-7-13 (single) were used for comparison. The results from *in vivo* MoMAb cocktail combinations varied slightly (66–100%) but in all cases resulted in protection of hamsters inoculated with canine RABV variants that was comparable to HRIG, independent of the concentration (400 IU/ml or 200 IU/ml) used (Table 5). In comparison to HRIG, the MoMAb cocktail combinations, adjusted to 2000 IU/10 ml, neutralized all but two lyssaviruses and the putative lyssavirus gts in *in vitro* studies (Table 6). Lyssaviruses not recognized by the mini-cocktail formulations were LBV and DUVV. In addition, MoMAb combination 62-7-13/62-7-13 was not able to neutralise EBLV-1.

**Table 5:** Protection of hamsters challenged with RABV following treatment with MoMAb combination cocktails during three independent *in vivo* studies.

MAb/MAb cocktail	Volume delivering 1000 IU/mL	400 IU/ml				200 IU/ml	
		Mex2004	Protection in %	Thai2006	Protection in %	India2008	Protection in %
PBS		n.d.		n.d.		5/9	
62-7-13/E559	0.34/0.45	8/9	88	6/9	66	7/9	77
62-7-13/M777	0.34/0.06	9/9	100	9/9	100	8/9	88
62-7-13/M727	0.34/0.01	8/9	88	6/9	66	8/9	88
62-7-13/62-7-13	0.34/0.34	9/9	100	5/9	55	9/9	100
**Controls**							
62-7-13/1112-1	0.34/0.08	9/9	100	7/9	77	n.d.	
62-7-13 only	0.34	9/9	100	1/9	11	n.d.	
HRIG positive control		4/9	44	5/9	55	7/9	77
HRIG negative control		3/9		0/9		3/9	

Survivorship of hamsters after challenge with a Mexican (2004), Thai (2006), or Indian (2008) canine RABV variant and subsequent treatment with 1∶1 cocktail formulations of MoMAbs to simulate passive immunization in PEP.

**Table 6:** *In vitro* neutralization pattern of equal amounts of MoMAbs in combination cocktails.

Virus	gt	Virus dose (log10)	Incubation time (days)	SRIG	PBS	62-7-13/E559	62-7-13/M777	62-7-13/M727	62-7-13/62-71-3
Bobcat, USA	1	4	2	0.125	+	0.125	0.125	0.125	0.125
Dog, Azerbaizhan	1	4	2	0.25	+	0.25	0.25	0.25	0.25
Dog, Ethiopia	1	4	2	0.125	+	0.25	0.5	0.25	2.00
Dog, India	1	4	2	1.00	+	0.125	0.125	0.5	0.5
Dog, Mexico	1	4	2	0.5	+	0.25	0.25	0.25	0.5
Dog, Nepal	1	4	2	0.25	+	0.25	0.25	0.25	0.5
Dog, Turkey	1	4	2	0.125	+	0.125	0.25	0.25	0.25
Fox, Eastern Europe	1	4	2	0.5	+	0.125	0.125	0.125	0.5
Fox, Europe	1	4	2	0.125	+	0.25	0.125	0.25	2.00
Polar fox, Norway	1	4	2	0.5	+	0.125	0.125	0.125	0.25
Wolf, Bosnia	1	4	2	0.125	+	1.00	1.00	1.00	1.00
EBLV-1, Germany	5	4	2	1.00	+	0.5	0.25	0.5	
EBLV-2, UK	6	4	2	2.00	+	0.5	0.5	1.00	1.00
Arctic, Canada	1	2	7	n.d.	+	0.009	0.005	0.009	0.018
Big Brown Bat, Canada	1	2	7	n.d.	+	0.002	0.005	0.003	0.026
CVS-11	1	2	7	n.d.	+	0.013	0.005	0.004	0.005
Dog, Sri Lanka	1	2	7	n.d.	+	0.007	0.014	0.009	0.026
ERA	1	2	7	n.d.	+	0.013	0.019	0.012	0.204
Mongoose, Africa	1	2	7	n.d.	+	0.009	0.014	0.009	0.026
Silver Haired Bat, Canada	1	2	7	n.d.	+	0.007	0.007	0.006	0.013
Vampire Bat, Latin America	1	2	7	n.d.	+	0.013	0.054	0.035	0.051
LBV, Africa	2	2	7	n.d.	+	+	+	+	+
DUVV, Africa	4	2	7	n.d.	+	+	+	+	+
EBLV-1, Europe	5	2	7	n.d.	+	0.019	0.014	0.035	+
EBLV-2, Europe	6	2	7	n.d.	+	0.003	0.027	0.009	0.036
ABLV, Australia	7	2	7	n.d.	+	0.007	0.003	0.004	0.005

*In vitro* neutralization pattern of equal mixes of MoMAbs in combination cocktails adjusted to 2000 IU/10 ml in comparison to SRIG against lyssaviruses of gt 1–7 and putative lyssavirus gts. Figures in boxes show the minimum MoMAb concentration in IU/ml at which complete neutralization was observed. Boxes with cross (+) represent the presence of viable virus.

## Discussion

Appropriate mixtures of RABV-specific MAbs generated *in vitro* would be a superior alternative to currently employed HRIG and ERIG for human PEP in rabies endemic areas [16]. Despite the fact that human hybridomas have been developed [40], the number of fully characterized HuMAb cocktails suitable for rabies PEP is still limited [27]–[29]. Mouse MAbs offer the next best alternative to HRIG and ERIG since they are able to completely neutralize RABV and their specific neutralizing activity (IUs per mg protein) is as much as 2,000 times higher than that of commercial HRIG [25],[36]. Here, we report for the first time, the identification of three novel combinations of MoMAbs that have a similar efficacy to HRIG and hence, could form the basis for an alternative to HRIG or ERIG.

Suitable candidate MoMAbs that form the basis of the individual cocktails were selected on the basis of stringent criteria, such as biological activity, neutralizing potency, binding specificity, spectrum of neutralization of natural lyssaviruses, and history of hybridomas, as applied for a HuMAb cocktail described recently [27]. These are the requirements for the development of safe and efficacious MAb alternatives to currently used polyclonal serum products [41]. The histories of the selected mouse hybridomas are well documented (Table 1). Alternative biologicals for PEP including MoMAbs have to overcome a number of problems associated with the hybridomas, including stability and contamination with potential pathogens [22]. From the information on the cell culture history for each MoMAb, the relative risk of contamination of the hybridomas with FMDV and TSE or and other adventitious agents was considered minimal because, in all cases, only approved fetal calf serum originating from countries free of FMD and TSE was used. Also, concerns associated with HuMAbs arising from the possibility of a potential spread of known and unknown human pathogens from hybridoma cells can be ignored with mouse hybridoma cells. One approach to overcome the problem of possible pathogen contamination of the MAb preparations, while maintaining the high binding affinities produced by somatic mutation of the B cell in response to antigenic stimulation, however, is to clone and express the immunoglobulin genes from the monoclonal hybridoma cell line in heterologous systems [22],[42],[43].

The production stability observed after 30 cell passages using serum-containing medium was satisfactory and met the requirements, as none of the mouse hybridomas of the candidate MoMAbs showed a considerable loss of VNA secretion. However, the optimal supernatant harvest time, taking into account cell viability, can still be optimised. Production of candidate MoMAbs under GLP conditions, as undertaken by a WHO service manufacturer, showed that the hybridoma yields could be improved at least 10–20 times in bioreactors (Table 4). However, the use of serum-free medium resulted in a decrease in the VNA production. Hence, adaptation to an appropriate commercially available serum-free medium should be the subject of further investigation. Based on experience, serum-containing medium might be preferential for conservation of the hybridomas, as this will result in a better and more stable survival rate of the hybridoma cells due to the protective function of the fetal calf serum. Isotyping showed the candidate MoMAbs to be of the IgG isotype (subtypes 1 & 2) and thus, ideal with respect to PEP, since IgGs are expected to have a longer half-life *in vivo* than other immunoglobulin types [44].

Our *in vitro* efficacy studies of candidate MoMAb demonstrated their capacity to neutralize a broad spectrum of RABV and other lyssaviruses of phylogroup I (Table 3), consistent with previous studies demonstrating cross-neutralization and cross-protection [1],[45]. The lack of cross-neutralization with phylogroup II viruses and WCBV was also expected from the phylogenetic distance, which correlates with previous studies [1],[38]. However, this limitation is less important as human infections with these genotypes are extremely rare and do not represent a major threat for public health. Previous studies comparing the antigenic phenotype of diverse RABV isolates showed that different neutralizing epitopes were shared between Pitman Moore (PM) and other RABV strains and supports our observations [46],[47]. However, none of the candidate MoMAbs alone was able to neutralize all of the RABVs tested. One explanation might be the different virus dose used in the *in-vitro* neutralisation assays, since higher concentrations of MoMAbs were needed to neutralize the higher virus dose (Table 3 and 6). On the other hand, RABV strains that were not neutralized *in vitro* may represent natural escape mutants if individual candidate MoMAbs were unable to recognize specific epitopes on the G. In contrast, a cocktail comprising all candidate MoMAbs conferred protection in the same model (data not shown). These data emphasise the need for an ideal therapeutic modality to consist of a mixture of at least two MoMAbs to ensure that all known RABV strains are targeted with a standardized reagent [16],[22],[48].

In addition to the broad spectrum of virus neutralization that these MAbs, in general, are capable of, candidate MoMAbs should target distinct, non-overlapping epitopes and should not compete for binding to the RABV G. Of the selected MoMAbs, all but one recognized antigenic site II on the RABV G, as shown by sequencing the epitope binding sites at the G-gene level and the generation of MoMAb-specific escape mutants in combination with *in vitro* cross-neutralization assays (Table 2). This indicated that MoMAb 62-7-13 (CDC, USA) was an essential component for a unique standardized MoMAb cocktail combination. Since one candidate MoMAb had to be excluded from further consideration because of intellectual property ownership, this resulted in three novel combinations of MoMAbs cocktails targeting non-overlapping epitopes present in antigenic sites II and III (Table 6).

Further characterization of the functional properties of the three unique MoMAbs cocktail combinations and their capacity to prevent the spread of RABV both *in vitro* and in animal models will be assessed in future studies. As these MoMAbs cocktail combinations can be considered as an alternative to HRIG and ERIG for PEP treatment in developing countries, the principal focus of the *in vitro* and *in vivo* studies remains on neutralization of RABVs isolated from dogs from different geographical areas ([4], Tables 3 and 6). In the blind *in vitro* efficacy studies of the three MoMAb cocktails, a range of doses was determined in an effort to determine the appropriate doses that could be used in animal studies. In animals, antibody administration may range from 2–4 IU per animal for small-bodied species, 200–400 IU per medium-size animals and up to 2000–4000 IU per large animal (or human). The actual formulations may be ∼150–200 IU/ml or more. However, *in vivo*, these rarely reach >1 IU/ml of serum. Thus, the range was considered optimal from ∼0.6 to 2.5 IU/ml. The MoMAb cocktail combinations showed neutralisation within the suggested IU range (Table 6). *In vivo*, all three MoMAb cocktail combinations resulted in protection rates (66–100%) for hamsters challenged with canine RABV variants that were comparable to HRIG (Table 5). Similar observations were made recently with other single MoMAbs depending on the strain of the virus [25]. As in this study, the *in vivo* testing was undertaken for ’proof-of-concept’. Clearly, further studies should be undertaken to provide additional statistical evidence.

Our preliminary *in vivo* studies with MoMAbs cocktails provided encouraging results. However, it could be presumed that the use of such antibodies in humans might have limitations, as with ERIG, because of the potential of foreign proteins to cause side effects. Even antibody fragments, which are less likely to be recognized as foreign could present problems as they seem less stable *in vivo* than whole antibodies [17]. Human MAbs for rabies PEP would be preferential; however, MoMAbs can be readily humanized [49]. Also, their unknown compartmentalization, half-life as well as immunogenicity in humans, is supposed to prevent MoMAbs from being ideal replacements for the existing reagents [16]. Despite these limitations, the end-product of the WHO project would be of an improved quality over ERIG.

The approach used here to develop and establish a suitable MoMAb cocktail combination of a minimum of two anti-G MoMAbs able to replace RIG for human PEP against rabies was based on the ultimate goal to make a product, which can be used in developing countries. Although the WHO project should be seen within the context of wider biological product market competitiveness, the ’uniqueness’ of the WHO project as described here is in the preferential conditions under which the product would have to be produced and made available to the public sector of rabies-endemic countries, particularly of the developing world (e.g. production costs). Therefore, the three novel MoMAb combination cocktails can be considered a less expensive alternative for prophylactic use to prevent rabies in humans. Currently, both phase I safety trials of the MoMAb product and humanization of the MoMAbs are under consideration.
