# Correction: Development of a Mouse Monoclonal Antibody Cocktail for Post-exposure Rabies Prophylaxis in Humans

**DOI:** 10.1371/annotation/df98339d-6bdb-40ed-af83-cc38b249264a

**Published:** 2009-11-10

**Authors:** Thomas Müller, Bernhard Dietzschold, Hildegund Ertl, Anthony R. Fooks, Conrad Freuling, Christine Fehlner-Gardiner, Jeannette Kliemt, Francois X. Meslin, Richard Franka, Charles E. Rupprecht, Noël Tordo, Alexander I. Wanderler, Marie Paule Kieny

Richard Franka was not included in the author byline. He should be listed as the ninth author, and his affiliation is 7: WHO Collaborating Centre for Reference and Research on Rabies, Rabies Section, Division of Viral and Rickettsial Diseases, Viral and Rickettsial Zoonoses Branch, National Center for Infectious Diseases, Centers for Disease Control and Prevention, Atlanta, Georgia, United States of America. The contributions of this author are as follows: Performed the experiments, analyzed the data, contributed reagents/materials/analysis tools.

